# Research Topics and Trends in MIMIC‐IV: A Large ICU Database Relevant for Critical Care Nursing

**DOI:** 10.1111/nicc.70440

**Published:** 2026-03-26

**Authors:** Yuh‐Shan Ho, Ahmed Ben Salem, Mahdi Kchaou, Abdulhameed Dere, Yosra Mzid, Houcemeddine Turki

**Affiliations:** ^1^ CT HO Trend Taipei City Taiwan; ^2^ Department of Radiology Habib Bourguiba University Hospital, University of Sfax Sfax Tunisia; ^3^ University of Sfax Sfax Tunisia; ^4^ College of Health Sciences, University of Ilorin Ilorin Nigeria

**Keywords:** biomedical informatics, clinical database, MIMIC‐IV, research topics, Web of Science Core Collection

## Abstract

**Background:**

The Medical Information Mart for Intensive Care‐IV (MIMIC‐IV) clinical database has become a central resource for data‐driven critical care research, enabling advances in clinical informatics, machine learning and nursing science. Despite its rapid uptake, no prior study has provided a transparent, methodologically grounded, bibliometrics‐based overview of MIMIC‐IV‐related research output.

**Aim:**

This paper aims to map the major research themes associated with the MIMIC‐IV database (2021–2024) and to evaluate their relevance to critical care nursing research and practice.

**Study Design:**

A study of 1150 publications retrieved from the Web of Science Core Collection (SCI‐Expanded). Explicit search strategies, front‐page filtering and publication counts were used to identify and analyse keyword‐based research themes.

**Results:**

Keyword analyses identified mortality prediction, sepsis, acute kidney injury, intensive care workflows and machine learning as dominant research areas, many of which are directly relevant to nursing‐sensitive outcomes and bedside clinical decision‐making.

**Conclusions:**

This review provides the first focused mapping of research themes within MIMIC‐IV publications. These findings clarify the thematic landscape of current MIMIC‐IV‐based research and underscore topics of particular importance to critical care nursing.

**Relevance to Clinical Practice:**

MIMIC‐IV supports the generation of evidence on essential nursing concerns. Recognising global research patterns enables nurses, clinicians and informatics teams to identify emerging tools, prioritise data‐driven competencies and translate large‐scale analytics into improved ICU care and patient outcomes.

## Impact Statements

- What is known about the topic
  ○ MIMIC‐IV is widely used in critical care research, particularly for studying outcomes such as mortality, sepsis and acute kidney injury through data‐driven methods.
  ○ Previous studies have demonstrated the value of large ICU databases for developing predictive models and supporting clinical decision‐making.
  ○ Although some MIMIC‐IV research addresses nursing‐sensitive outcomes and clinical workflows, these areas have not been systematically mapped.
- What this paper adds
  ○ This study provides the first focused scoping review of MIMIC‐IV publications (2021–2024), identifying major research themes and trends.
  ○ It demonstrates the strong relevance of dominant MIMIC‐IV research topics to critical care nursing practice and bedside decision‐making.
  ○ The findings offer a structured evidence base to guide nurses, clinicians and informatics teams in prioritising future data‐driven research and competencies.

**Abbreviations**
AF: atrial fibrillation
AI: artificial intelligence
AKI: acute kidney injury
AP: acute pancreatitis
AUC: area under the curve
BERT: bidirectional encoder representations from transformers
BMI: body mass index
BUN: blood urea nitrogen
COPD: chronic obstructive pulmonary disease
CVD: cardiovascular disease
EHRs: electronic health records
ePVS: estimated plasma volume status
HF: heart failure
HGI: haemoglobin glycation index
ICU: Intensive Care Unit
IMV‐LSTM: interpretable multi‐variable long short‐term memory
KNN: K‐nearest neighbours
LASSO: least absolute shrinkage and selection operator
LightGBM: light gradient boosting machine
LIME: local interpretable model‐agnostic explanations
LOS: length of stay
LSTM: long short‐term memory
MI: myocardial infarction
MIMIC‐IV: Medical Information Mart for Intensive Care‐IV
ML: machine learning
NLR: neutrophil‐to‐lymphocyte ratio
PLR: platelet‐to‐lymphocyte ratio
RDW: red cell distribution width
RF: random forest
SAPS II: Simplified Acute Physiology Score II
SHAP: SHapley Additive exPlanations
SHR: stress hyperglycaemia ratio
SII: Systemic Immune‐Inflammation Index
SOFA: Sequential Organ Failure Assessment
SpO_2_
: peripheral oxygen saturation
SQL: structured query language
SVM: support vector machine
TCN: temporal convolutional networks
Transformer: transformer (neural network architecture)
TyG: triglyceride–glucose
XGBoost: extreme gradient boosting

## 1 Introduction

The digitisation of healthcare has produced large volumes of clinical data that support advances in medical research, nursing practice and patient care [1]. Electronic health records (EHRs) are now central to observational studies, predictive modelling and clinical decision support. Among publicly available resources, the MIMIC‐IV database has become a major dataset, offering deidentified ICU data such as vital signs, labs, interventions and clinical notes [2]. These elements are particularly relevant to critical care nursing, which depends on continuous monitoring, interpretation of physiologic trends and timely decision‐making.

MIMIC‐IV extends earlier MIMIC versions and provides a high‐resolution dataset for studying ICU outcomes, developing machine learning models and examining disease progression [2, 3]. Its breadth has supported research in mortality prediction, sepsis, acute kidney injury, cardiovascular disorders and other analytics‐driven domains aligned with nursing priorities such as early deterioration detection, complication prevention and optimising care interventions.

As research using MIMIC‐IV accelerates, understanding the dataset's scientific influence is increasingly important [4]. Examining publication trends, citation patterns and thematic focus areas helps characterise its academic impact and identify opportunities to strengthen evidence‐based practice through clinical data [5, 6]. This is especially valuable in critical care nursing, where high‐quality data support patient safety, refined assessments and advanced bedside analytics.

This review analyses the bibliographic metadata of MIMIC‐IV research indexed in Web of Science's SCI‐Expanded to characterise research topics and their relevance to data‐driven medicine and ICU nursing. We first describe the MIMIC‐IV database (Section 2), explain our analytical approach (Section 3), present and discuss findings (Section 4) and conclude with future directions for MIMIC‐IV research and this review (Section 5).

## 2 Background

Medical Information Mart for Intensive Care‐IV (MIMIC‐IV) is a comprehensive, deidentified electronic health record (EHR) dataset developed by MIT and released through PhysioNet. Available as a relational SQL database and via Google BigQuery, it enables efficient querying with standard SQL tools [7]. The dataset includes information from thousands of patients admitted between 2008 and 2019, covering vital signs, laboratory results, diagnoses, procedures, medications and free‐text clinical notes [2]. Its scope supports diverse research applications, from predictive modelling to clinical workflow analysis.

MIMIC‐IV is organised into four major modules: Core, Hospital, ICU and Emergency Department (ED), each representing different components of patient care. The core module contains demographics and admission details, the hospital module includes diagnoses and procedures, the ICU module provides high‐resolution time‐series data and the ED module captures emergency encounters. These modules are linked through standard identifiers (subject_id for patients, hadm_id for hospital admissions, icustay_id for ICU stays), allowing integrated, longitudinal analyses across care settings [2].

As an open database, MIMIC‐IV facilitates transparent, reproducible research and serves as a key resource for developing clinical prediction models, studying disease trajectories and evaluating interventions [7]. It has supported numerous studies on outcomes such as mortality and sepsis, underscoring its significant role in advancing data‐driven critical care research [4].

## 3 Study Design and Methods

A study of 1150 publications retrieved from the Web of Science Core Collection (SCI‐Expanded). Explicit search strategies, front‐page filtering, and publication counts were used to identify and analyse keyword‐based research themes.

### 3.1 Data Source and Search Strategy

**FIGURE 1:**
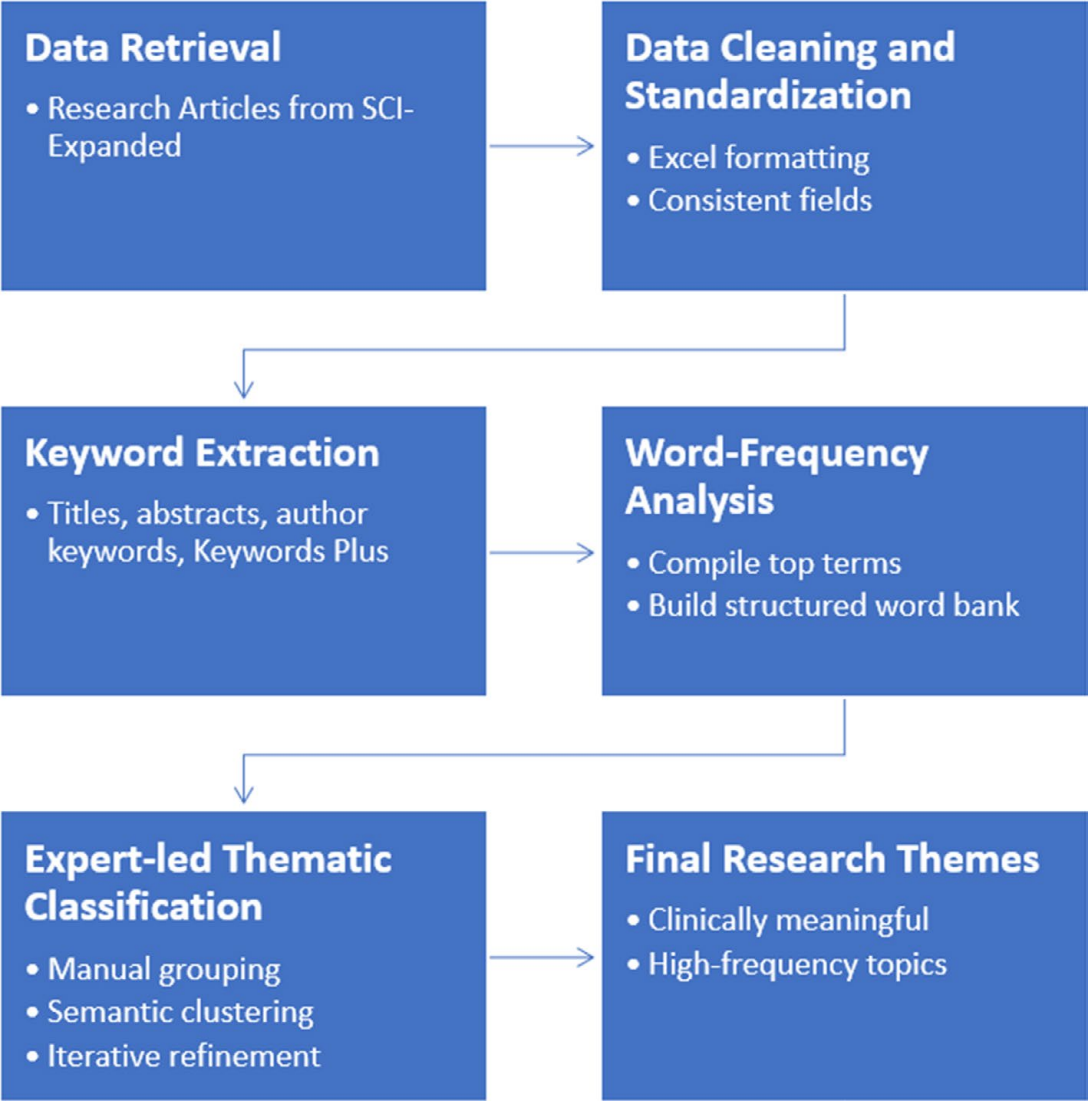
Process for the identification of the MIMIC‐IV research themes.

This review employed a structured bibliometric approach to systematically identify, screen and analyse publications related to the MIMIC‐IV clinical database. The methodology comprised three main stages: data retrieval from the Web of Science Core Collection's Science Citation Index Expanded (SCI‐EXPANDED), eligibility screening and filtering and thematic classification of research topics based on bibliographic metadata. An overview of the approach is presented in Figure 1, while the detailed procedures are provided in the following subsections.

### 3.2 Topic, Scope and Eligibility

This review analysed publications related to the MIMIC‐IV clinical database using records retrieved from the SCI‐EXPANDED database in the Web of Science Core Collection (WoSCC). Searches were conducted in the Topic (TS) field, which includes title, abstract, author keywords and Keywords Plus, for publications from 2021 to 2024.

The exact WoS query was:

*TS = (‘MIMIC‐IV’ OR ‘MIMIC IV’ OR ‘Medical Information Mart for Intensive Care IV’ OR ‘Medical Information Mart for Intensive Care (MIMIC) IV’)*.

**FIGURE 2:**
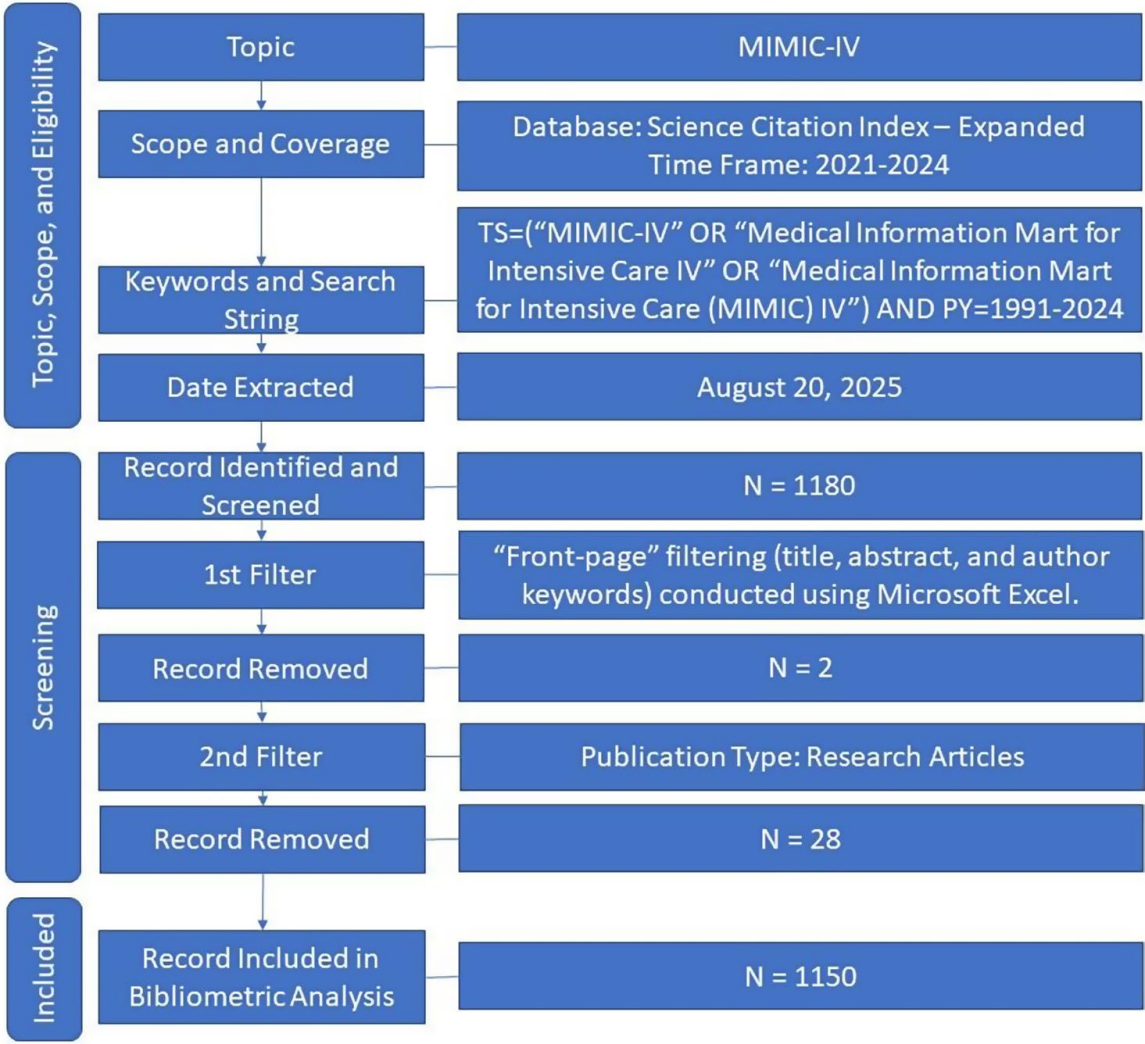
Flow chart for the search process.

Eligible document types included articles, reviews, early‐access items and conference papers. All data were extracted on 20 August 2025. A PRISMA‐style workflow is shown in Figure 2.

The Web of Science, specifically SCI‐EXPANDED, was selected as the sole source database for this analysis due to its strength in indexing high‐impact, interdisciplinary research across the sciences, social sciences and, crucially, the multidisciplinary domain of biomedical informatics where much MIMIC‐IV research is published [8]. Its curated citation index and consistent indexing practices support robust bibliometric and citation‐based analyses, which are central to this review's aims of mapping influential research themes and trends [9]. While databases such as PubMed offer excellent coverage of clinical and nursing literature [10], SCI‐EXPANDED provides a focussed lens on the broader, citation‐active scientific conversation around a complex resource like MIMIC‐IV, which attracts significant contributions from computational, informatics and engineering fields alongside clinical disciplines [8]. We acknowledge that this choice introduces a potential coverage bias, as some nursing‐specific or clinically focused journals may be less represented in SCI‐EXPANDED than in PubMed or Scopus [10]. Consequently, the findings may under‐represent certain practice‐orientated nursing studies [11]. However, for the purpose of identifying dominant, cross‐disciplinary research themes and the trajectory of a technically intensive dataset like MIMIC‐IV within the broader scientific literature, Web of Science offers a strategically appropriate and methodologically consistent scope [8]. This approach enhances transparency by clearly defining the corpus as the set of MIMIC‐IV studies embedded within the mainstream, citation‐linked scientific record.

### 3.3 Screening

The initial search retrieved 1180 records. A front‐page filter [12, 13] was applied, retaining records in which ‘MIMIC‐IV’ or its variants appeared in the title, abstract or author keywords. This OR‐based logic avoids overly restrictive filtering and helps remove false positives, especially those retrieved only through *Keywords Plus* (e.g., due to the generic term *mimic*).

Two records were excluded because the term ‘mimic’ appeared only in the Keywords Plus field. Non‐research items (e.g., reviews, editorials, letters) were then removed. After filtering, 1150 research articles remained, as shown in Figure 2.

### 3.4 Data Processing and Standardisation

Bibliographic records were downloaded in plain‐text format and processed in Microsoft Excel 365 [14, 15].

Research themes were identified using word‐frequency analysis of titles, abstracts, author keywords and Keywords Plus [5, 6]. Most common keywords across all kinds of bibliographic metadata were compiled as a word bank. Then, the top keywords featured in the titles or as author keywords are identified and grouped to constitute research themes. The word bank as well as the retrieved top keywords are made available as Supporting Information to this manuscript. This frequency‐based descriptive approach was selected over keyword co‐occurrence or network analysis to align with the review's primary aim of providing a transparent, high‐level mapping of the dominant research landscape. While co‐occurrence analysis excels at revealing relational structures and clusters among concepts [16], the frequency method offers a more direct and interpretable overview of the most prominent, standalone topics within the corpus, which is essential for identifying broad, nursing‐relevant themes.

The grouping of high‐frequency terms into coherent research themes was performed manually by domain experts in critical care and nursing informatics. This expert‐led, qualitative clustering approach, consistent with principles of qualitative content analysis, was chosen to prioritise clinical relevance and interpretive validity over purely algorithmic grouping. Experts reviewed the term list and iteratively grouped related terms into thematic categories based on semantic meaning and their relevance to critical care practice. Thematic saturation was assessed through this iterative process; clustering continued until no new substantive categories emerged and all high‐frequency terms were meaningfully accounted for within the established thematic framework. This method ensures that the resulting themes are not only data‐driven but also clinically meaningful and actionable for nursing.

## 4 Results

Keyword analyses identified mortality prediction, sepsis, acute kidney injury, intensive care workflows, and machine learning as dominant research areas, many of which are directly relevant to nursing‐sensitive outcomes and bedside clinical decision‐making.

A total of 1150 MIMIC‐IV‐related articles were analysed to identify research foci. Author keywords were available in 90% of the publications, providing a strong basis for theme extraction. Excluding search terms, Table 1 lists the top 20 author keywords and title words. From 2021 to 2024, terms such as ‘mortality’, ‘sepsis’, ‘intensive care unit’, ‘machine learning’ and ‘acute kidney injury’ were consistently dominant. The frequent appearance of ‘machine learning’ and ‘artificial intelligence’ reflects broader trends in computer science, where large clinical datasets increasingly rely on deep learning and data‐driven methods [17, 18].

**TABLE 1:** The 20 most frequently used words in the title and author keywords.

Words in the title	TP	Rank (%)	Author keywords	TP	Rank (%)
Patients	865	1 (75)	Mortality	279	1 (27)
Mortality	534	2 (46)	Sepsis	223	2 (22)
Retrospective	349	3 (30)	Intensive care unit	169	3 (16)
Acute	299	4 (26)	Machine learning	123	4 (12)
Association	283	5 (25)	Acute kidney injury	120	5 (12)
Database	276	6 (24)	Prognosis	105	6 (8.3)
Ill	227	7 (20)	In‐hospital mortality	86	7 (8.0)
Critically	225	8 (20)	Nomogram	83	8 (6.8)
Cohort	223	9 (19)	Critical care	70	9 (4.8)
Sepsis	182	10 (16)	All‐cause mortality	50	10 (4.7)
Injury	177	11 (15)	ICU	49	11 (3.7)
Kidney	167	12 (15)	Prediction model	38	12 (3.2)
In‐hospital	163	13 (14)	Heart failure	33	13 (3.1)
Ratio	148	14 (13)	Acute myocardial infarction	32	14 (3.0)
Prediction	144	15 (13)	Atrial fibrillation	31	15 (2.9)
Risk	136	16 (12)	Acute pancreatitis	30	16 (2.8)
Learning	134	17 (12)	28‐day mortality	29	17 (2.7)
Machine	128	18 (11)	Septic shock	28	17 (2.7)
Unit	126	19 (11)	Prediction	28	19 (2.6)
ICU	117	20 (10)	Critically ill patients	27	20 (2.5)

Abbreviations: TP: number of articles containing the keywords; %: percentage in each category.

**FIGURE 3:**
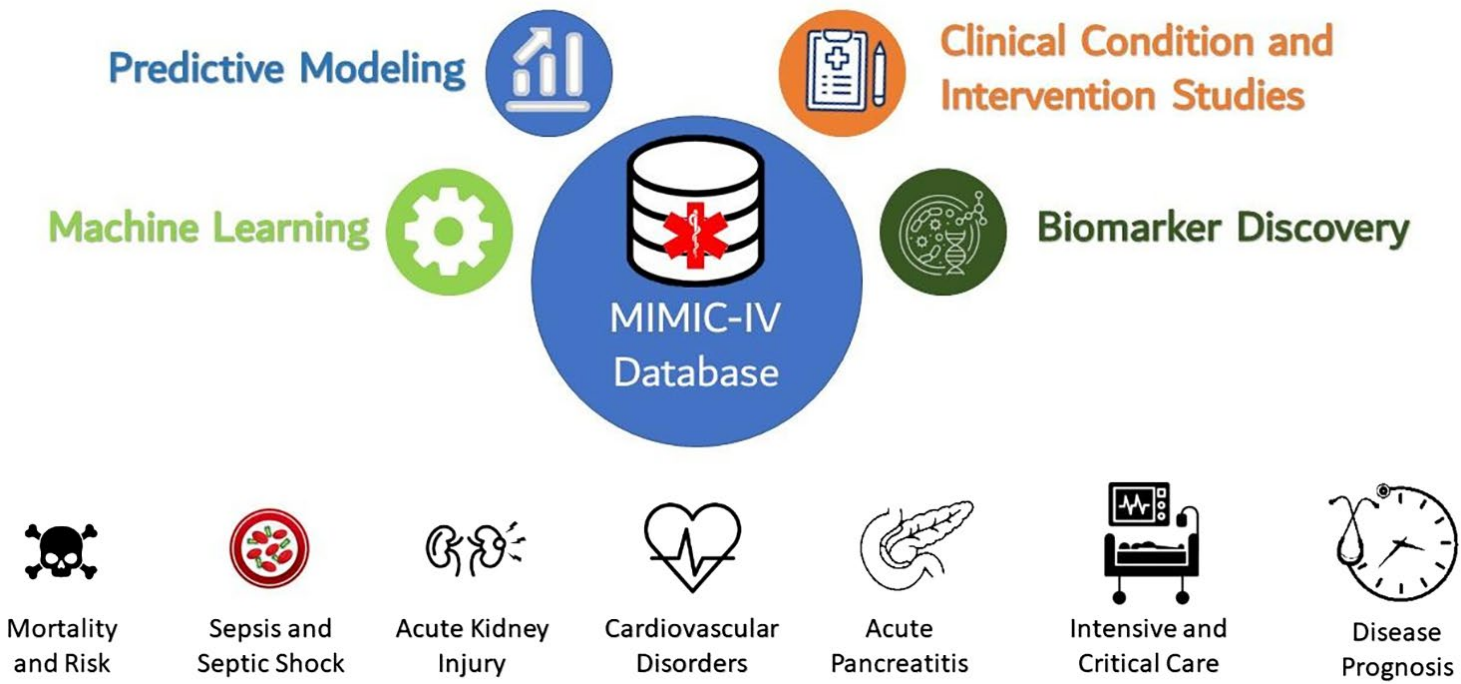
Major research themes about MIMIC‐IV.

The major research themes formed by these keywords are shown in Figure 3 and align with prior findings in open medical database research [4], biomedical data mining [19], clinical natural language processing [20], bioinformatics [21] and AI in biomedicine [22, 23, 24]. The critical care nursing translation of these research themes is outlined in Table 2.

**TABLE 2:** Translating MIMIC‐IV research themes into critical care nursing competencies.

Major research theme	Key insights from MIMIC‐IV studies	Relevant critical care nursing competencies	Practice implications for nursing
Mortality prediction and risk stratification	Identifies robust biomarkers (e.g., TyG index, SHR, NLR) and models for prognosticating outcomes in sepsis, CVD and stroke.	*Physiologic surveillance and interpretation*: Advanced monitoring of metabolic and inflammatory trends. *Clinical judgement and risk assessment*: Integrating biomarker data into dynamic patient risk profiling.	Enables nurses to identify high‐risk patients earlier, tailor monitoring intensity and prioritise care interventions based on data‐driven risk scores.
Sepsis and septic shock	Evaluates prognostic factors, optimal timing for interventions (e.g., vasopressin) and efficacy of therapies (e.g., aspirin, anticoagulation).	*Protocol development and implementation*: Applying evidence to sepsis bundle adherence and resuscitation protocols. *Interprofessional decision support*: Collaborating on timely intervention based on predictive analytics.	Empowers nurses as key agents in early detection, protocol‐driven management and team‐based titration of therapies to improve sepsis outcomes.
Acute kidney injury (AKI)	Models for predicting AKI onset and progression, links to sepsis, pancreatitis, and drug exposures (e.g., contrast, antibiotics).	*Surveillance for complications*: Recognising early signs of renal injury in high‐risk populations. *Medication and fluid management*: Assessing nephrotoxic risk and optimising fluid balance based on predictive insights.	Guides preventive nursing actions, such as vigilant monitoring of at‐risk patients and advocating for nephroprotective strategies.
ICU workflows and outcome prediction	Machine learning models predict LOS, mortality and complications; algorithms process high‐volume time‐series data.	*Informatics literacy*: Understanding the logic, limitations and outputs of clinical prediction models. *Workflow optimisation*: Using predictive data to anticipate discharge needs or clinical deterioration.	Helps nurses interpret AI‐generated alerts, participate in model refinement and use predictions to streamline care planning and resource allocation.
Cardiovascular disorders	Prognostic insights for AF, HF and MI focussing on metabolic control, inflammation and frailty.	*Advanced haemodynamic monitoring*: Interpreting complex data (e.g., arrhythmia burden, volume status) in cardiac patients. *Patient education and transition planning*: Counselling on long‐term risk factors identified during ICU stay.	Supports nurses in managing cardiac instability and providing targeted education based on individualised risk profiles identified in the ICU.
Acute pancreatitis (AP)	Identifies prognostic markers (e.g., lactate‐albumin ratio, TyG index) for mortality; highlights associations with AKI, sepsis and multi‐organ dysfunction.	*Metabolic and inflammatory surveillance*: Monitoring trends in lactate, glucose and inflammatory markers. *Complication prevention and management*: Anticipating and managing AKI, sepsis and nutritional deficits specific to AP.	Enables early recognition of patients at risk for severe AP or organ failure, guiding vigilant monitoring, timely fluid resuscitation and coordinated care with nutritional and gastrointestinal specialists.
Models and algorithms	Development and validation of interpretable AI tools (e.g., nomograms, ML pipelines) for clinical decision support.	*Data‐informed practice*: Critically appraising model recommendations in the clinical context. *Interprofessional communication*: Articulating AI‐derived insights within the care team.	

The following sections summarise the state of the art within each theme, grounded in the bibliometric evidence from the 2021–2024 corpus. Several 2025 studies are referenced only to contextualise these trends and clarify ongoing methodological and clinical developments; they are not part of the analysed dataset and do not extend the review's publication window.

## 5 Discussion

The findings highlight that MIMIC‐IV research integrates clinical biomarkers, physiologic trends, and machine learning models to inform precision risk stratification, supporting proactive, data‐driven critical care nursing interventions.

### 5.1 Mortality Prediction, Risk Stratification and Disease Prognosis

Recent studies leveraging the MIMIC‐IV database have established the triglyceride–glucose index (TyG‐i) and its composite measure, TyG‐BMI, as promising predictors of mortality among critically ill patients across diverse cardiovascular and metabolic conditions [25, 26]. Lower TyG‐BMI levels were associated with significantly higher 1‐year all‐cause mortality in heart failure patients [26], higher TyG index values correlated with increased in‐hospital and ICU mortality in sepsis [27], and predicted higher mortality across all follow‐up intervals in haemorrhagic stroke [28]. Hu et al. extended these findings to atrial fibrillation, showing an ‘L‐shaped’ inverse relationship between TyG‐BMI and mortality at multiple timepoints [29]. These data suggest that TyG‐based metrics serve as accessible, non‐invasive biomarkers integrating lipid–glucose metabolism and insulin resistance for prognostic risk stratification in critical care. The prognostic significance of TyG‐derived indices converges in critical illness but uncovers heterogeneity in their directional associations, protective in cardiac dysfunction yet deleterious in sepsis and stroke. This implies disease‐specific metabolic dynamics and differential roles of insulin resistance across pathophysiological contexts. Current research gaps include the absence of prospective validation as well as limited and unclear cut‐off thresholds for clinical application.

Studies leveraging the MIMIC‐IV database have demonstrated the prognostic value of the *stress hyperglycaemia ratio* (SHR) in critically ill populations across cardiovascular and cerebrovascular contexts. Elevated SHR has been independently associated with short‐ and long‐term all‐cause mortality [30]. Across diverse cohorts, including atrial fibrillation [31], acute myocardial infarction, coronary heart disease [32, 33, 34] and cerebrovascular disease [35], high SHR values predicted greater ICU, in‐hospital, and 1‐year mortality, often displaying *U‐ or J‐shaped* associations with survival. Several analyses identified that non‐diabetic patients were vulnerable to stress hyperglycaemia–related mortality. Most studies applied Cox proportional hazards models, logistic regression, restricted cubic spline analyses and Kaplan–Meier survival curves, reinforcing the robustness of findings. Literature converges on SHR as a reproducible, non‐invasive biomarker linking stress‐induced hyperglycaemia to adverse outcomes in ICU patients. However, heterogeneity exists regarding optimal cut‐off values.

The current literature positions delirium as a prognostic biomarker. Studies provide converging evidence that delirium significantly increases short‐term mortality and interacts with key physiological markers. Zhang et al. [36] developed a predictive model identifying five independent mortality predictors in patients with sepsis or sepsis‐associated delirium [36]. Complementarily, Liu et al. analysed 22 361 older adult ICU patients and found delirium in approximately 24% of cases, confirming it as an independent predictor of in‐hospital mortality after adjustment via propensity score matching [37]. Logistic regression revealed significant interactions between delirium, SOFA score and haemoglobin levels, with mortality risk attenuated in patients with SOFA > 12 or haemoglobin > 15 g/dL. Together, these studies underscore the complex interplay between delirium, organ dysfunction, and metabolic derangements in critically ill populations [37].

Emerging evidence from MIMIC‐IV‐based studies highlights the prognostic role of systemic inflammatory markers. NLR, PLR and SII were independently associated with all‐cause mortality in patients with atrial fibrillation [38]. These findings support the use of simple haematological ratios as cost‐effective biomarkers for mortality risk stratification in AF. Complementarily, Jiang et al. investigated 16 007 septic ICU patients and found a J‐shaped association between SII and 28‐day mortality, with both low and high SII values conferring elevated risk [39]. These studies establish inflammation‐derived indices as robust predictors of adverse outcomes, reflecting immune dysregulation and systemic stress in critical illness.

Recent MIMIC‐IV‐based studies have explored other diverse prognostic biomarkers emphasising metabolic, haemodynamic and inflammatory pathways. Glycaemic variability predicts poorer consciousness and higher in‐hospital mortality among traumatic brain injury patients [40]. Similarly, the haemoglobin glycation index (HGI) [41] demonstrated a U‐shaped association with mortality in critically ill coronary artery disease patients, underscoring the prognostic relevance of dysregulated glucose metabolism. In cardiovascular settings, estimated plasma volume status (ePVS) and frailty scores emerged as independent predictors of short‐ and long‐term mortality in myocardial infarction and heart failure, with frailty notably enhancing predictive models beyond conventional risk scores [42]. In sepsis, renal mean perfusion pressure [43] and organism type [44] were strongly linked to outcomes, while timely vasopressin initiation [45] and aspirin therapy [46] showed protective associations, improving survival in septic shock and sepsis‐associated acute kidney injury, respectively. Additionally, serum ionised calcium [47] displayed a U‐shaped relationship with ischaemic stroke mortality, reinforcing the importance of electrolyte balance. Collectively, these investigations highlight the prognostic potential of routinely measurable clinical and laboratory parameters, promoting precision stratification in critical care through real‐world evidence. However, heterogeneity in measurement definitions, retrospective design, and limited external validation restrict clinical translation. The analysis of the MIMIC‐IV research outputs reveals a growing interest in non‐conventional biomarkers, underscoring the field's transition toward individualised pathophysiology‐driven models of investigation.

Collectively, this body of MIMIC‐IV‐derived evidence contributes to critical care nursing knowledge by demonstrating that routinely available metabolic, inflammatory, neurologic and haemodynamic biomarkers function as integrated indicators of physiologic stress, reserve and dysregulation rather than isolated laboratory abnormalities. These findings inform nursing assessment by supporting continuous, trend‐based physiologic surveillance that integrates glucose dynamics, lipid–metabolic signals, mental status changes, inflammatory burden and perfusion parameters to identify early, disease‐specific deterioration patterns. At the level of ICU shifts, this evidence reinforces nurses' role in anticipatory clinical decision‐making, prioritising high‐risk patients for intensified monitoring, timely escalation, and targeted interventions, while highlighting the need for contextual interpretation of biomarkers rather than reliance on single threshold values.

### 5.2 Critical Illness Management and Implications for Critical Care Nursing

To provide a comprehensive perspective on how MIMIC‐IV‐derived evidence informs prognosis, intervention strategies and nursing practice in critical care, findings from disease‐specific and system‐level investigations are synthesised across Tables 3 and 4. Together, these syntheses trace a progression from empirically derived predictors of adverse outcomes to their implications for time‐sensitive clinical judgement, nursing competencies and the advancement of critical care nursing knowledge.

**TABLE 3:** MIMIC‐IV‐derived biomarkers, interventions and predictive approaches across critical illness domains.

Disease domain	Indicator/intervention/model	Outcome or prognostic value	Key references
General intensive and critical care	ICU length‐of‐stay models	Predict recovery trajectory and resource utilisation	[48]
	Global ICU mortality models	Support early deterioration recognition	[49, 50]
	Ketamine exposure	Associated with outcomes in ventilated patients	[51]
	BMI and delirium	Identify neurologic complication risk	[52]
Sepsis and septic shock	Prophylactic heparin	Early anticoagulation reduces sepsis mortality	[53]
	Initial ventilation strategy	Influences in‐hospital mortality	[54]
	Triglyceride–Glucose (TyG) Index	Independent predictor of in‐hospital mortality, sepsis‐associated AKI and prolonged hospitalisation	[27, 55, 56]
	Systemic Immune‐Inflammation Index (SII)	High‐fidelity inflammatory marker predicting mortality	[39]
	Vasopressin initiation timing	Time‐dependent determinant of survival in septic shock	[45]
	Dynamic vasoactive medication trends	Enables real‐time mortality risk prediction using ML	[57]
	Aspirin exposure	Associated with improved outcomes in sepsis‐induced myocardial injury and AKI	[46, 58]
Acute kidney injury (AKI)	Glycaemic variability	Independent predictor of ICU 30‐day mortality	[59]
	Pulse wave velocity	Reflects haemodynamic stress influencing AKI outcomes	[60]
	TyG Index	Associated with AKI severity, length of stay and sepsis‐related outcomes	[55]
	Serum calcium and magnesium	Predict AKI onset in cirrhotic and acute pancreatitis patients	[61, 62]
	Ondansetron exposure	Associated with reduced AKI mortality	[63]
	Early AKI prediction models	Predict AKI development within 7 days of ICU admission	[64]
Cardiovascular disorders	Stress hyperglycaemia ratio	Predicts all‐cause mortality in critically ill AF patients	[31]
	TyG‐BMI	Strong predictor of 1‐year mortality in AF and heart failure	[26, 29]
	Serum anion gap	Independent prognostic factor in myocardial infarction	[65]
	EASIX score	Marker of endothelial dysfunction linked to AF mortality	[66]
	Influenza vaccination	Modifiable factor reducing AF mortality	[67]
	Frailty indices	Predict short‐ and long‐term mortality in HF and MI	[68, 69]
	Machine‐learning mortality models	Improve risk stratification in MI and HF‐AF populations	[70, 71]
Acute pancreatitis (AP)	Lactate‐albumin ratio	Superior predictor of 28‐day mortality	[72]
	Bilirubin‐to‐albumin ratio	Associated with short‐ and long‐term mortality	[73, 74]
	RDW‐to‐albumin ratio	Reflects inflammation–nutrition interaction	[75]
	TyG Index	Predicts disease severity and sepsis risk	[76, 77]
	Laboratory‐based frailty index	Quantifies physiologic reserve and mortality risk	[78]

**TABLE 4:** Integrated mapping of MIMIC‐IV non‐biomarker evidence to critical care nursing practice and learning outcomes.

Dimension/nursing learning outcome	Core MIMIC‐IV insight	Relevance to critical care nursing practice	Educational/conceptual implication	Key references
Therapeutic timing and time‐critical intervention awareness	Clinical outcomes depend more on *when* interventions are initiated than on treatment exposure alone	Reinforces nurse vigilance for time‐sensitive therapies, escalation and protocol adherence	Emphasises narrow therapeutic windows and urgency in nursing decision‐making	[45, 54]
Dynamic physiologic trends and early deterioration recognition	Longitudinal physiologic trends outperform single measurements for predicting mortality and organ failure	Supports continuous monitoring, trend‐based assessment and early escalation by nurses	Strengthens early warning systems and shift‐level prioritisation skills	[36, 57, 79]
Machine‐learning‐supported clinical decision‐making	ML models improve prediction of mortality, respiratory failure and AKI	Enables nurse‐informed use of decision‐support and risk alerts	Enhances evidence‐guided decision‐making under uncertainty	[64, 70, 71]
Frailty, physiologic reserve and risk stratification	Functional reserve and frailty strongly modify outcomes across diagnoses	Encourages holistic assessment beyond organ‐specific parameters	Guides care prioritisation and monitoring intensity	[68, 69, 78]
Multisystem pathophysiology and systems‐based assessment	Critical illness reflects interacting metabolic, inflammatory, renal and hepatic dysfunction	Promotes integrated, systems‐based nursing surveillance	Reinforces holistic ICU assessment frameworks	[39, 55, 73]
Preventive care and patient safety	Vaccination, anticoagulation and glucose stability reduce mortality risk	Highlights nursing roles in prevention, adherence and safety initiatives	Aligns with quality improvement and patient safety competencies	[53, 59, 67]
Shift‐level risk evolution and prioritisation	Patient risk evolves over hours rather than days	Validates nurse‐led reassessment, prioritisation and rapid escalation	Supports dynamic workload and acuity management models	[50, 57]
Precision‐informed nursing care	Individualised risk stratification improves targeting of monitoring and interventions	Strengthens nursing contributions to personalised critical care	Advances precision nursing and data‐driven care models	[49, 50]
Interdisciplinary collaboration	Data‐driven insights support shared clinical decision‐making	Enhances nurse–physician communication and coordinated care	Reinforces team‐based, data‐informed ICU practice	[57, 70]

The synthesis presented in Table 3 integrates metabolic and inflammatory biomarkers, therapeutic exposures, and predictive modelling approaches associated with mortality, organ dysfunction and other adverse outcomes across major critical illness domains, including sepsis and septic shock, acute kidney injury, cardiovascular conditions, acute pancreatitis and heterogeneous ICU populations. Rather than isolating individual predictors, this body of evidence illustrates how routinely collected physiologic, laboratory and treatment data converge to characterise high‐risk clinical trajectories. Across conditions, adverse outcomes emerge not from singular abnormalities but from the interaction of metabolic stress, inflammatory burden, physiologic reserve and the timing of therapeutic interventions. These relationships are discernible through the continuous and high‐resolution data captured within MIMIC‐IV.

Complementing these biomarker‐focussed findings, the integrated synthesis in Table 4 broadens the analytic lens to include non‐biomarker dimensions of critical illness and their relevance to nursing practice and education. This evidence highlights the importance of therapeutic timing, longitudinal physiologic trends, machine learning–enabled risk stratification, multisystem pathophysiology, preventive and modifiable exposures and the evolution of patient risk over the course of hours rather than days. Collectively, these insights emphasise that critical illness is inherently dynamic and process‐driven, shaped as much by the temporal pattern of physiologic change and clinical response as by the presence of derangement itself.

When situated within a nursing framework, these findings underscore the centrality of continuous surveillance, trend interpretation and timely escalation of care. The alignment of MIMIC‐IV evidence with core nursing learning outcomes demonstrates how large‐scale ICU data analytics directly support essential practice domains, including early recognition of clinical deterioration, systems‐based physiologic assessment, risk stratification and prioritisation, precision‐informed monitoring, prevention and patient safety and interdisciplinary collaboration. In this context, nurses emerge not merely as data recipients but as critical interpreters and integrators of evolving physiologic, metabolic and functional information at the bedside.

Taken together, this synthesis illustrates how MIMIC‐IV research advances critical care by linking biomarker discovery, therapeutic processes and nursing practice within a unified, data‐informed framework. The convergence of metabolic and inflammatory indicators, dynamic physiologic trends, treatment timing and predictive modelling supports a shift away from static, diagnosis‐centred assessment toward continuous, systems‐oriented and precision‐informed care. For critical care nursing, these insights reinforce the value of leveraging real‐time and longitudinal data to anticipate deterioration, prioritise interventions, and contribute meaningfully to interdisciplinary clinical decision‐making in the intensive care unit.

### 5.3 Models and Algorithms for Data Processing

Research leveraging the MIMIC‐IV database employs a diverse array of modelling frameworks and algorithmic strategies, encompassing both deep learning interpretability approaches and traditional machine learning pipelines. Meng et al. [80] developed an MIMIC‐IV‐based framework to evaluate model interpretability (i.e., the extent to which a model's predictions can be understood by humans) and fairness (i.e., the extent to which algorithms avoid discriminatory behaviour towards specific individuals or groups) across deep neural architectures, including LSTM, Transformer, temporal convolutional networks (TCN) and IMV‐LSTM, trained on time‐series and static clinical data for mortality prediction. Their interpretability assessment integrates post hoc methods such as Integrated Gradients, DeepLIFT and SHAP, while fairness is quantified through subgroup bias metrics across demographic attributes. This dual focus on explainability and fairness distinguishes MIMIC‐IF as a meta‐framework for evaluating clinical AI transparency rather than a single predictive model. Complementing this interpretability‐driven research, Gupta et al. [81] developed a modular MIMIC‐IV data processing pipeline that standardises cohort construction, imputation, temporal feature extraction and model evaluation, serving as a generalisable foundation for subsequent predictive modelling efforts.

Building upon these foundational pipelines, numerous clinical prediction studies using MIMIC‐IV have applied machine learning models to forecast outcomes such as acute kidney injury [64, 82], in‐hospital mortality [49, 50, 83] and length of ICU stay [48]. These studies typically follow a structured data processing pipeline: cleaning and merging time‐series tables, performing feature engineering and selection (often via random forest importance or recursive elimination), and comparing multiple algorithms such as logistic regression, random forest, support vector machines, gradient boosting and neural networks. Interpretability remains central to these works, with SHAP and LIME dominating as feature attribution techniques that help validate model findings against known physiological indicators. Röhr et al. [84] and others have further contributed benchmark frameworks that emphasise data harmonisation and reproducibility across predictive modelling pipelines, highlighting the importance of consistent preprocessing for fair model comparison. Table 5 summarises these diverse modelling strategies, identifying each review's algorithms, feature processing techniques and interpretability tools.

**TABLE 5:** Examples of machine learning‐based approaches for processing MIMIC‐IV data.

Study	Clinical task/outcome	Main algorithms/models	Feature processing and selection	Interpretability/fairness methods
Meng et al. [80]	General ICU outcome prediction; interpretability and fairness	LSTM, transformer, temporal convolutional networks, IMV‐LSTM	Data truncation, data aggregation, missing‐value imputation, normalisation, categorical encoding	Integrated Gradients, DeepLIFT, SHAP, fairness gap metrics
Gupta et al. [81]	Data pipeline design for MIMIC‐IV	Modular ML pipeline (supports random forest, logistic regression, gradient boosting, XGBoost, recurrent neural network, LSTM, temporal neural network and transformers)	Missing‐value imputation, temporal feature extraction, cohort filtering	Modular interpretability hooks (e.g., ROC‐based metrics and fairness modules)
Lin et al. [64]	Acute kidney injury in acute pancreatitis	Random forest, SVM, KNN, neural networks, linear model, naive Bayes, gradient boosting	Feature selection via RF importance, scaling	Feature importance analysis
Tian et al. [82]	Acute kidney injury in liver cirrhosis	Random forest, XGBoost, LightGBM, gradient boosting decision tree	Univariate selection, clinical feature curation	Feature importance analysis and ROC‐based model comparison
Sun et al. [50]	ICU cardiac arrest mortality	logistic regression, LASSO, XGBoost	Stepwise regression, multi‐collinearity checks	Nomogram
Röhr et al. [84]	Benchmarking clinical outcome prediction	BERT	Unified pre‐processing benchmark for MIMIC‐IV	AUC values per category
Hempel et al. [48]	ICU length of stay	XGBoost, random forest, SVM, logistic regression	Feature aggregation of vitals/labs	Feature importance analysis + partial dependence
Pang et al. [83]	ICU mortality	XGBoost, logistic regression, SVM, decision tree	Feature selection based on ROC curve‐based metrics like AUC	Feature importance analysis + SHAP
Lin et al. [71]	30‐day mortality in myocardial infarction	XGBoost, random forest, logistic regression	Feature selection based on ROC curve‐based metrics like AUC	Feature importance analysis + nomogram
Wu et al. [85]	Delirium in older adult COPD patients	Random forest, XGBoost, logistic regression, SVM	Feature selection based on LASSO regression and the best subset method	SHAP global and local interpretation
Li et al. [49]	In‐hospital mortality in acute heart failure	random forest, XGBoost, SVM, KNN, decision trees	Feature selection based on LASSO regression	Feature importance analysis + calibration curves
Han et al. [86]	Sepsis‐associated encephalopathy	XGBoost, LightGBM, CatBoost, multilayer perceptron, SVM	Feature selection based on LASSO regression and Boruta methods	SHAP summary plots
Xie et al. [87]	In‐hospital death in severe diabetic ketoacidosis	XGBoost, logistic regression, Bayesian information criterion	Multivariate logistic regression filtering	Feature importance analysis + nomogram
Hu et al. [88]	Sepsis‐associated liver injury	Multivariate logistic regression (LASSO)	Multi‐collinearity screening	Nomogram
Shi et al. [89]	Diabetic Ketoacidosis prolonged ICU stay	Logistic regression (LASSO)	Feature selection via multivariate analysis	Nomogram

In simple terms, researchers using the MIMIC‐IV database follow similar steps when building prediction models. First, they clean and organise large amounts of patient data. Then, they use computer algorithms to look for patterns that can predict outcomes such as death, kidney injury or length of stay in intensive care. Many studies compare several types of models to find the most accurate one. Importantly, researchers also use special tools to explain how these models make decisions and to check that they do not treat certain patient groups unfairly. This focus on transparency and fairness helps ensure that AI systems can be trusted and understood in clinical practice.

A substantial subset of MIMIC‐IV studies has employed *nomogram‐based logistic regression models* to enhance clinical interpretability while retaining statistical rigour. Lin et al. [71] constructed a nomogram predicting 30‐day mortality among myocardial infarction patients, achieving strong discriminative power (AUC = 0.835) with key predictors such as age, BUN and SpO_2_. Similarly, Hu et al. [88] developed a LASSO‐selected logistic regression nomogram for sepsis‐associated liver injury mortality (AUC = 0.809), outperforming SOFA and SAPS II scores, whereas Peng et al. [90] applied comparable methods to myocardial infarction mortality. These models emphasise calibration, discrimination and clinical usability through visual decision aids. Table 6 details these nomogram frameworks, summarising their key variables, validation metrics and cohort characteristics. Together, these studies reveal that MIMIC‐IV's data processing ecosystem, spanning deep learning interpretability frameworks, modular ML pipelines and nomogram‐based logistic models, balances computational complexity with clinical transparency, forming a robust methodological backbone for critical care outcome prediction.

**TABLE 6:** Examples of nomogram‐based approaches driven by MIMIC‐IV.

Study/outcome	Model type	Key predictors/feature categories	Validation metrics/performance	Cohort/sample notes
Lin et al. [71]: 30‐day mortality in critical MI	Nomogram built from logistic regression (after ML screening)	Age, blood urea nitrogen, heart rate, SpO_2_, bicarbonate, metoprolol use (among others)	In validation set: AUC = 0.835 (95% CI 0.774–0.897); good calibration; accuracy ~0.914 versus SOFA score AUC 0.735	Patients with myocardial infarction admitted to CCU, extracted from MIMIC‐IV
Hu et al. [88]: in‐ICU mortality in older adults with sepsis‐associated liver injury	Nomogram based on LASSO + multivariate logistic regression	Six final variables identified (e.g., bilirubin, INR, others)	Training set AUC = 0.814, validation set AUC = 0.809; calibration curves; decision curve analysis; compared versus SAPS II (0.798) and SOFA (0.634)	Older adult ICU patients with sepsis and liver injury (defined by Total Bilirubin > 2 mg/dL, INR > 1.5) from MIMIC‐IV; n_training = 653, n_validation = 281
Peng et al. [90]: in‐hospital mortality in Myocardial Infarction	Nomogram (logistic regression)	Classical myocardial infarction predictors	Performance metrics reported and validated (AUC, calibration) in the internal validation cohort	Retrospective cohort from MIMIC‐IV, ~4688 MI patients

Collectively, this body of MIMIC‐IV‐based modelling research advances critical care nursing knowledge by demonstrating that robust outcome prediction can be achieved when advanced machine learning, deep learning and traditional statistical frameworks are paired with transparency, fairness and clinical interpretability. These findings inform nursing assessment and physiologic surveillance by validating that routinely collected time‐series data (vital signs, laboratory trends and organ function markers) contain actionable signals of deterioration that can be made interpretable and clinically meaningful through explainable AI and nomogram‐based tools. At the level of ICU shifts, this evidence supports nursing clinical decision‐making by reinforcing trust in data‐driven risk stratification, enabling earlier recognition of high‐risk trajectories, and facilitating nurse engagement with predictive outputs that align with physiologic reasoning rather than opaque black‐box predictions.

### 5.4 Limitations

The evidence is largely retrospective, with variable biomarker definitions, limited external validation, gaps in ICU workflow, nursing‐sensitive outcomes, and multimodal data, and is further constrained by the exclusive use of Scopus, which may have omitted relevant studies indexed in other databases.

## 6 Relevance to Clinical Practice

MIMIC‐IV supports the generation of evidence on essential nursing concerns. Recognising global research patterns enables nurses, clinicians, and informatics teams to identify emerging tools, prioritise data‐driven competencies, and translate large‐scale analytics into improved ICU care and patient outcomes.

## 7 Conclusions

This review provides the first focused mapping of MIMIC‐IV‐based research (2021–2024) using SCI‐Expanded data, revealing a clear division between mature, well‐established themes and emerging areas with substantial growth potential. Mature domains are characterised by high publication volume, methodological standardisation, and strong clinical relevance. These include mortality prediction and risk stratification using biomarkers and machine learning; sepsis and septic shock research addressing prognostic markers, therapies, and organ failure prediction; acute kidney injury modelling across critical conditions; cardiovascular disorders such as atrial fibrillation, heart failure and myocardial infarction; and data processing approaches that commonly employ standardised machine learning pipelines, interpretability tools (e.g., SHAP, LIME) and nomogram‐based regression models.

In contrast, several areas remain underdeveloped and present important opportunities for future, particularly nurse‐led, research. These include ICU workflows and care processes, nursing‐sensitive outcomes, patient experience and family‐centred care, health equity and disparities, intervention effectiveness and implementation science and the integration of multimodal data such as nursing notes and physiological waveforms. Addressing these gaps would shift MIMIC‐IV research beyond prediction towards practical, equitable and translational insights. Future work should therefore balance refinement of established models with exploratory studies that directly inform nursing practice, patient safety and care quality at the bedside.

## Author Contributions

Y.‐S.H., A.B.S. and H.T. conceptualised the work. Y.‐S.H. did the data collection. Y.‐S.H., A.B.S., M.K., A.D. and H.T. did the investigation and wrote the main manuscript text. Y.‐S.H., A.B.S., Y.M., and H.T. reviewed the manuscript and supervised the research work.

## Funding

The authors have nothing to report.

## Ethics Statement

The authors have nothing to report.

## Consent

The authors have nothing to report.

## Conflicts of Interest

The authors declare no conflicts of interest.

## Data Availability

The retrieved word bank, together with word frequencies from titles and author keywords, is publicly available as Supporting Information at https://doi.org/10.5281/zenodo.18662383.
